# S100A4 released from highly bone-metastatic breast cancer cells plays a critical role in osteolysis

**DOI:** 10.1038/s41413-019-0068-5

**Published:** 2019-09-23

**Authors:** Haemin Kim, Bongjun Kim, Sang Il Kim, Hyung Joon Kim, Brian Y. Ryu, Junho Chung, Zang Hee Lee, Hong-Hee Kim

**Affiliations:** 1 Department of Cell and Developmental Biology, BK21 Program and DRI, Seoul National University, Seoul, Korea; 2 Department of Biochemistry and Molecular Biology, Seoul National University College of Medicine, Seoul, Korea; 3 Cancer Research Institute, Seoul National University College of Medicine, Seoul, Korea; 4 Department of Cancer Biology, Seoul National University College of Medicine, Seoul, Korea; 5 Department of Oral Physiology, BK21 PLUS Project, and Dental and Life Science Institute, School of Dentistry, Pusan National University, Yangsan, Korea; 6 Interdisciplinary Program in Bioinformatics, Seoul National University College of Natural Sciences, Seoul, Korea; 7 Department of Biomedical Sciences, Seoul National University Graduate School, Seoul, Korea

**Keywords:** Bone cancer, Homeostasis

## Abstract

Bone destruction induced by breast cancer metastasis causes severe complications, including death, in breast cancer patients. Communication between cancer cells and skeletal cells in metastatic bone microenvironments is a principal element that drives tumor progression and osteolysis. Tumor-derived factors play fundamental roles in this form of communication. To identify soluble factors released from cancer cells in bone metastasis, we established a highly bone-metastatic subline of MDA-MB-231 breast cancer cells. This subline (mtMDA) showed a markedly elevated ability to secrete S100A4 protein, which directly stimulated osteoclast formation via surface receptor RAGE. Recombinant S100A4 stimulated osteoclastogenesis in vitro and bone loss in vivo. Conditioned medium from mtMDA cells in which S100A4 was knocked down had a reduced ability to stimulate osteoclasts. Furthermore, the S100A4 knockdown cells elicited less bone destruction in mice than the control knockdown cells. In addition, administration of an anti-S100A4 monoclonal antibody (mAb) that we developed attenuated the stimulation of osteoclastogenesis and bone loss by mtMDA in mice. Taken together, our results suggest that S100A4 released from breast cancer cells is an important player in the osteolysis caused by breast cancer bone metastasis.

## Introduction

Bone metastasis affects >80% of patients with breast cancer at advanced stages of the disease and causes severe pain, fracture, hypercalcemia, and nerve compression, leading to increased morbidity and mortality.^1,2^ Most bone metastases of breast cancer are catabolic to the skeleton. The osteolytic nature of breast cancer bone metastasis is attributed to a vicious cycle between the tumor cells and the local bone environment.^1,2^ In this process, tumor cells that have metastasized to bone secrete factors such as parathyroid hormone-related peptide (PTHrP) and interleukin 8 to stimulate osteoclast formation either directly or indirectly via induction of receptor activator of nuclear factor (NF)-κB ligand (RANKL; the osteoclast differentiation factor) in osteoblasts.^3^ The consequent increase in bone resorption leads to the release of latent matrix-associated growth factors that enhance tumor growth. Among the released factors, transforming growth factor (TGF)-β plays a crucial role in the vicious cycle by upregulating PTHrP expression in tumor cells.^4^ In addition to their central role in the vicious cycle of bone destruction, osteoclasts have been proposed in recent studies to reactivate tumor cells from a dormant state, established after colonization in the bone microenvironment, to a proliferating state.^5,6^ Therefore, given our current understanding of the microenvironment, molecules that mediate communication between osteoclasts and tumor cells are attractive therapeutic targets for bone-metastatic cancers.

S100A4, a calcium-binding S100 family protein, has been characterized as an important regulator of cancer progression and metastasis.^7,8^ S100A4 has been shown to modulate the proliferation, apoptosis, motility, and invasiveness of various types of tumor cells. Several distinct mechanisms have been implicated in the molecular function of S100A4. For the cytoskeletal modulations required for cell motility, adhesion, and invasion, S100A4 interacts with non-muscle myosin II, liprin, and ezrin in the cytoplasm.^9–11^ To regulate apoptosis, S100A4 interacts with and suppresses the DNA-binding activity of the tumor-suppressor protein p53.^12^ In addition to these intracellular mechanisms, extracellular actions of S100A4 include the stimulation of neurite outgrowth, angiogenesis, matrix metalloproteinase (MMP) activity, and migration of astrocytic tumor cells.^13–16^ Although both intracellular and extracellular functions of S100A4 have been shown to be associated with tumor progression and metastasis, the precise mechanism of action of S100A4 remains elusive. In addition, the role of S100A4 in bone metastasis of breast cancers has not yet been reported.

In this study, we found that a highly bone-metastatic subline of MDA-MB-231 breast cancer cells released more S100A4 than the parental cells and had a greater stimulatory effect on osteoclastogenesis. The effect of S100A4 on osteoclasts was mediated by the cell surface receptor RAGE (receptor for advanced glycation end products). Furthermore, we provide in vivo evidence for the role of tumor-derived S100A4 in bone destruction and osteoclast formation by examining the effects of silencing the S100A4 gene and of a S100A4-blocking antibody (Ab) in a mouse xenograft model of breast cancer bone metastasis.

## Results

### Bone-metastatic breast cancer cells stimulate osteoclastogenesis in RANKL-dependent and RANKL-independent manners

**Fig. 1:**
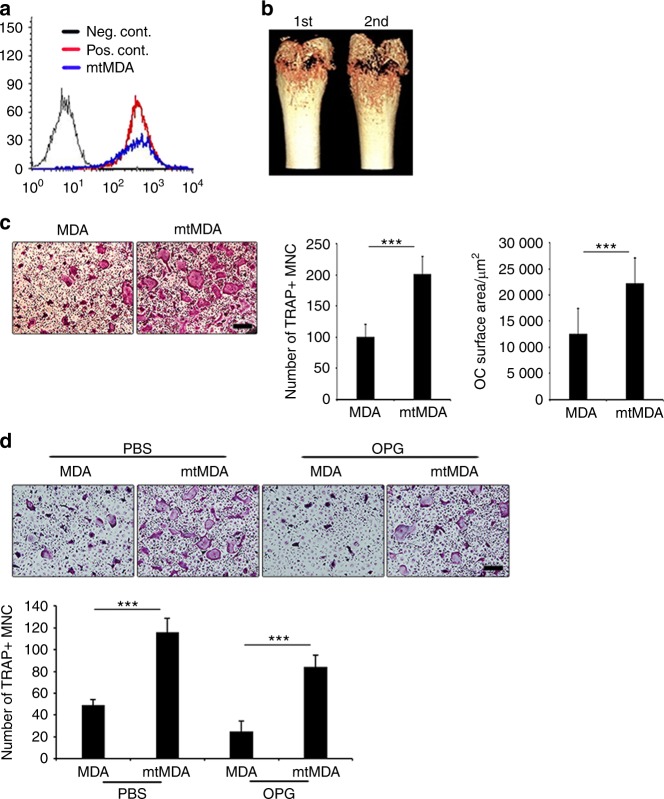
Bone-metastatic breast cancer cells induce osteolysis and stimulate osteoclastogenesis. **a** Flow cytometry with anti-human β2-microglobulin antibody verified mtMDA as human cells. Neg. Cont. mouse bone marrow cells, Pos. Cont. HEK-293 cells. **b** Microcomputed tomographic images of femurs showed more osteolysis after the second-round injection than the first-round injection. **c** More mature osteoclast (OC) formation (number of tartrate-resistant acid phosphatase-positive multinucleated cells (MNCs)) and greater OC surface area were observed in cultures that received mtMDA conditioned media (CM) than in cultures that received MDA CM. *n* = 4 per group (left) or 6 per group (right). ***P < 0.001 by unpaired two-tailed Student’s *t* test. **d** Addition of osteoprotegerin (100 ng·mL^–1^) partially inhibited the enhancement of OC formation by MDA and mtMDA. *n* = 5 per group. ***P < 0.001 by two-way analysis of variance with post hoc Tukey’s test. All data are presented as the mean ± SD. Scale bars, 200 μm

To obtain breast cancer cells with a high potency to metastasize to bone, we utilized a previously reported in vivo selection method.^17^ MDA-MB-231 (MDA) cells were injected into the left ventricle of immune-deficient Balb-c/nu mice. After 8 weeks, the tibiae and femurs were flushed, and the collected cells were cultured for 2 months to remove mortal mouse cells while selecting immortal cancer cells. These cells were then reinjected in the mice for another round of the selection process. The cancer cells after the second round were named mtMDA (highly bone-metastatic MDA). Flow cytometry with anti-human β2-microglobulin showed that the mtMDA cells were human and homogeneous (Fig. 1a), demonstrating that these cells were a subline of the MDA cells. Interestingly, microcomputed tomographic (μCT) analyses revealed more severe bone destruction in mice during the second round of selection than during the first round (Fig. 1b). Because this bone phenotype indicated that the mtMDA cells might be more osteolytic than the MDA cells, we compared the effects of the two cell types on osteoclastogenesis of mouse primary cells. Conditioned media (CM) prepared from mtMDA and MDA cells were added to pre-osteoclasts generated by priming bone marrow-derived macrophages (BMMs) with RANKL. The CM from mtMDA cells increased the number of mature tartrate-resistant acid phosphatase-positive (TRAP^+^) multinucleated cells (osteoclasts) and the osteoclast surface area compared with the CM from MDA cells (Fig. 1c). Since RANKL is an essential factor for osteoclastogenesis, we next tested whether osteoprotegerin (OPG), a decoy receptor of RANKL, could block the effect of the cancer cell CM. Surprisingly, OPG reduced osteoclast numbers by only 27.7% ± 5.14% in the culture with mtMDA CM and 49.3% ± 14.10% in the culture with MDA CM (Fig. 1d), indicating that RANKL was not the sole factor responsible for the stimulatory effect of these cancer cells on osteoclastogenesis.

### S100A4 is upregulated in bone-metastatic breast cancer cells

**Fig. 2:**
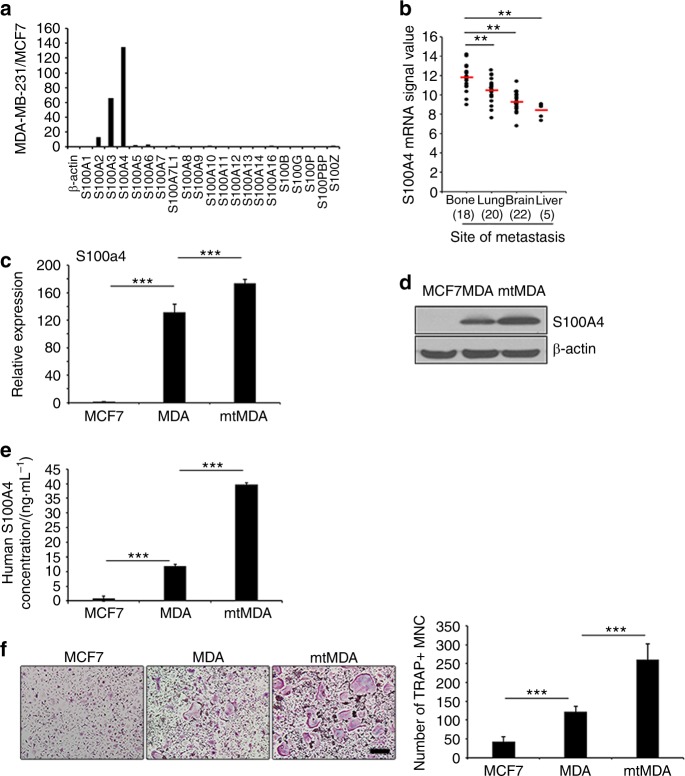
S100A4 expression and secretion is elevated in mtMDA cells. **a** A cDNA microarray revealed a higher signal intensity of S100A2, 3, and 4 in MDA than in MCF7 cells. **b** Human S100A4 signals retrieved from GSE 14020 of the GEO database from 65 breast cancer patients with the indicated sites of metastases. *n* = 18 (bone), 20 (lung), 22 (brain), and 5 (liver). **P < 0.01 by one-way analysis of variance (ANOVA) with post hoc Bonferroni test. **c** Real-time PCR analyses showed S100A4 mRNA expression in MDA and mtMDA but not in MCF7 cells. *n* = 3 per group. ***P < 0.001 by one-way ANOVA with post hoc Tukey’s test. **d** Western blot analyses showed that S100A4 protein expression was significantly higher in mtMDA than in MDA cells. **e** Enzyme-linked immunosorbent assay with conditioned media (CM) revealed the highest amount of S100A4 secretion from mtMDA and no secretion from MCF7. *n* = 3 per group. ***P < 0.001 by one-way ANOVA with post hoc Tukey’s test. **f** CM from mtMDA, MDA, and MCF7 stimulated osteoclast formation in decreasing order. *n* = 5 per group. ***P < 0.001 by one-way ANOVA with post hoc Tukey’s test. Data in **c**, **e**, and **f** are presented as the mean ± SD. Scale bars, 200 μm

To identify molecules associated with breast cancer bone metastasis, we analyzed the gene expression profiles of the bone-metastatic MDA and the nonmetastatic MCF7 breast cancer cell lines (Supplementary Fig. 1). Among the genes that were highly differentially expressed, the signal for S100A4 was 134.68-fold higher in the MDA than in the MCF7 cell line (Fig. 2a). Furthermore, a search of NCBI GEO microarray data sets of tumor samples from 65 breast cancer patients (GSE 14020) revealed a significantly higher expression of S100A4 in bone-metastasized tumors than in tumors metastasized to other organs (Fig. 2b). Using quantitative real-time PCR analyses, we confirmed a markedly higher level of S100A4 mRNA in the MDA cell line than in the MCF7 cell line. The S100A4 mRNA level was even higher in the mtMDA cells (Fig. 2c). A similar pattern was detected in intracellular S100A4 protein levels (Fig. 2d). We next examined S100A4 secretion by an enzyme-linked immunosorbent assay (ELISA) specific for human S100A4 protein with CM from cancer cells. The S100A4 concentration was 3.33-fold higher in mtMDA CM than in MDA CM, and MCF7 CM contained a negligible amount of S100A4 (Fig. 2e). Next, we explored the relationship between S100A4 levels in these cancer cells and the osteoclastogenic effects of cancer cell CM. As shown in Fig. 2f, MDA CM and mtMDA CM increased osteoclast formation by 2.9- and 6.2-fold, respectively, compared with MCF7 CM (Fig. 2f). Collectively, these results indicated that bone-metastasized breast cancer cells have high levels of S100A4 gene expression and protein secretion and that S100A4 secretion may be associated with cancer-induced osteoclastogenesis.

### S100A4 mediates bone-metastatic cancer-induced osteoclastogenesis

**Fig. 3:**
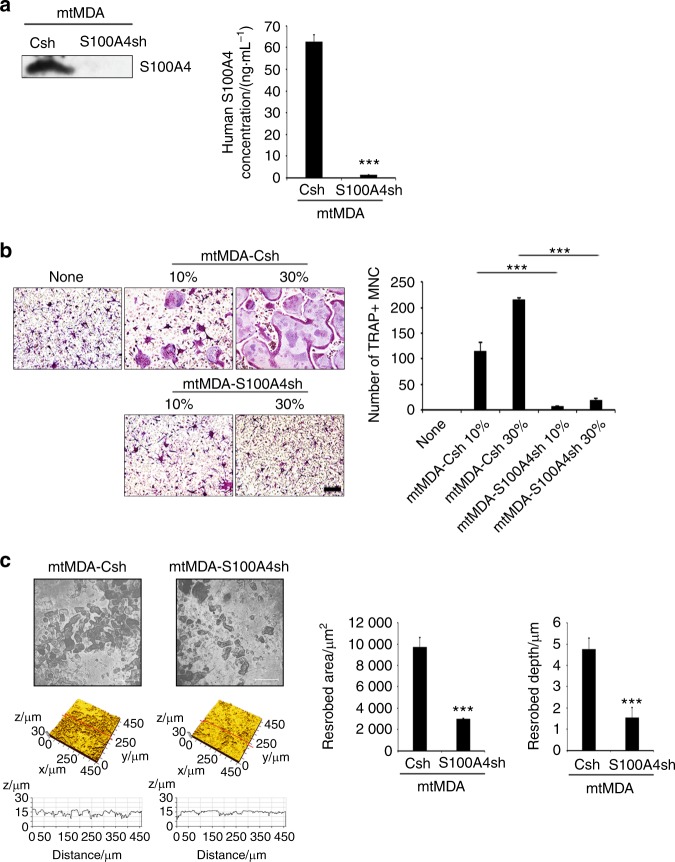
S100A4 secreted from mtMDA enhances osteoclastogenesis. **a** Lentiviral transduction of S100A4 short hairpin RNA (shRNA) (S100A4sh) efficiently reduced S100A4 protein levels in conditioned media (CM) from mtMDA (left, western blot; right, enzyme-linked immunosorbent assay). Csh control shRNA. *n* = 3 per group. ***P < 0.001 by unpaired two-tailed Student’s *t* test. **b** S100A4 knockdown nullified the osteoclastogenesis stimulatory effect by mtMDA CM. Representative images of tartrate-resistant acid phosphatase (TRAP)-stained cells (left) and quantification of TRAP^+^ multinucleated cells (right) are shown. *n* = 3 per group. ***P < 0.001 by one-way analysis of variance with post hoc Tukey’s test. **c** Bone resorption assays with dentine slices showed lower osteoclast activity in cultures with mtMDA-S100A4sh CM than in cultures with mtMDA-Csh CM. Representative confocal images of dentine surfaces (left) and values of resorbed area and depth of pits (right) are presented. *n* = 3 per group. ***P < 0.001 by unpaired two-tailed Student’s *t* test. All data are presented as the mean ± SD. Scale bars, 200 μm

Next, we investigated whether S100A4 was responsible for the stimulation of osteoclastogenesis by bone-metastatic breast cancer cells. To this end, we knocked down S100A4 in mtMDA cells by lentiviral transduction of shRNA. A successful reduction in S100A4 expression at both the mRNA and protein levels was verified by real-time PCR analyses and western blotting of whole-cell lysates (Supplementary Fig. 2). Western blot and ELISA analyses of culture supernatants showed an effective decrease in S100A4 secretion from the knockdown cells (Fig. 3a). We then tested the effect of CM from these cells on osteoclastogenesis. The CM from control knockdown cells (mtMDA-Csh) increased osteoclastogenesis as did mtMDA CM, while the CM from S100A4 knockdown cells (mtMDA-S100A4sh) had only a weak effect (Fig. 3b). Consistently, lower bone-resorbing activity was observed in mtMDA-S100A4sh-CM-treated cultures than in mtMDA-Csh-CM-treated cultures (Fig. 3c). A direct coculture of cancer cells and pre-osteoclasts also showed reduced osteoclast formation by S100A4 knockdown in mtMDA cells (Supplementary Fig. 3).

**Fig. 4:**
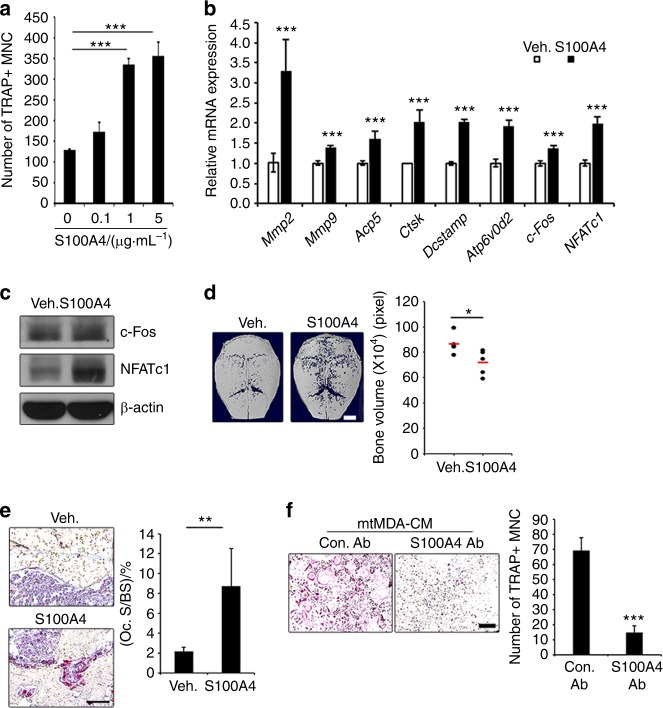
S100A4 directly promotes osteoclastogenesis. **a** Addition of rS100A4 protein increased mature osteoclast (OC) formation. *n* = 3 per group. ***P < 0.001 by one-way analysis of variance with post hoc Tukey’s test. **b** The mRNA levels of OC marker genes increased by treatment with mouse rS100A4 (1 μg·mL^−1^) for 1 day. *n* = 3 per group. ***P < 0.001 by unpaired two-tailed Student’s *t* test. **c** Western blots of c-Fos and NFATc1 in pre-OCs after treatment with mouse rS100A4 (1 μg·mL^−1^) for 24 h. **d** Microcomputed tomographic analysis of ICR mouse calvariae injected with vehicle (Veh.) or mouse rS100A4 every other day for 8 days. *n* = 5 per group. Representative images (left) and quantification of bone volume (right) are presented. *P < 0.05 by unpaired two-tailed Student’s *t* test. Scale bars, 2 mm. **e** Tartrate-resistant acid phosphatase-stained sections of calvarial bones from **d**. *n* = 5 per group. **P < 0.01 by unpaired two-tailed Student’s *t* test. Scale bars, 50 μm. **f** Blocking S100A4 function with anti-S100A4 Ab decreased osteoclastogenesis induced by conditioned media from mtMDA. *n* = 3 per group. ***P < 0.001 by unpaired two-tailed Student’s *t* test. Scale bars, 100 μm. All histogram data are presented as the mean ± SD

To more directly assess the effect of S100A4 on osteoclastogenesis, we next added mouse recombinant S100A4 protein (rS100A4) to osteoclast cultures. S100A4 increased the formation of TRAP^+^ multinucleated cells (Fig. 4a). Consistently, the mRNA expression of osteoclast differentiation marker genes such as MMP2/9, Acp5 (TRAP), cathepsin K (CtsK), DC-stamp, and Atp6v0d2 was significantly increased by S100A4 (Fig. 4b). The mRNA and protein levels of c-Fos and NFATc1, key transcription factors for osteoclastogenesis, were also increased (Fig. 4b, c). In addition, direct administration of rS100A4 protein onto mouse calvariae elicited calvarial bone lysis (Fig. 4d) and increased the percentage of osteoclast surface per bone surface (Oc.S/BS; Fig. 4e). To gain further evidence for the involvement of S100A4 in mtMDA CM-induced osteoclastogenesis, we utilized a commercial S100A4 blocking Ab. The addition of the S100A4 Ab to the mtMDA CM-treated culture strongly reduced osteoclast formation (Fig. 4f). Taken together, these data suggest that S100A4 secreted from mtMDA stimulates the generation of functional osteoclasts.

### S100A4 enhances osteoclastogenesis by stimulating canonical NF-κB via RAGE

**Fig. 5:**
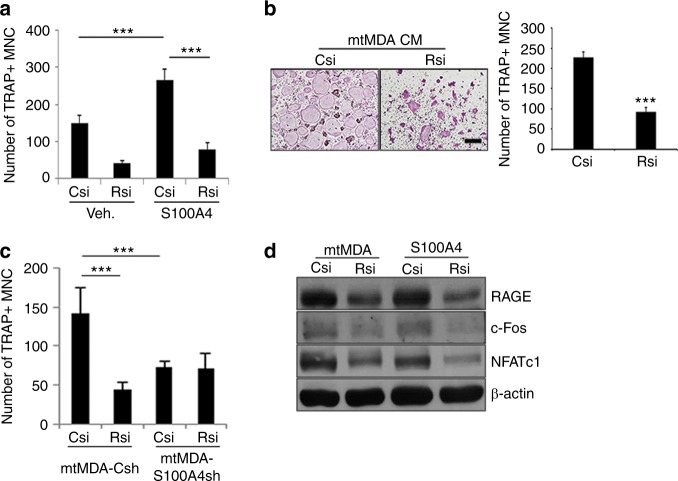
S100A4-induced osteoclastogenesis is mediated by RAGE (receptor for advanced glycation end products). **a** RAGE knockdown decreased S100A4-induced osteoclastogenesis. Pre-osteoclasts (pre-OCs) with either control (Csi) or RAGE (Rsi) knockdown were treated with vehicle (Veh.) or rS100A4 (1 μg·mL^−1^) for 2 days before tartrate-resistant acid phosphatase (TRAP) staining. TRAP^+^ multinucleated cells (MNCs) were counted. *n* = 5 per group. ***P < 0.001 by two-way analysis of variance (ANOVA) with post hoc Tukey’s test. **b** Pre-OCs with either Csi or Rsi were treated with mtMDA conditioned media (CM) for 2 days. TRAP^+^ MNCs were counted. *n* = 3 per group. ***P < 0.001 by unpaired two-tailed Student’s *t* test. Scale bars, 200 μm. **c** Pre-OCs with either Csi or Rsi were treated with CM from mtMDA-Csh or mtMDA-S100A4sh cells for 2 days. TRAP^+^ MNCs were counted. *n* = 5 per group. ***P < 0.001 by two-way ANOVA with post hoc Tukey’s test. **d** Pre-OCs with either Csi or Rsi were treated with mtMDA CM or S100A4 (1 μg·mL^−1^) for 1 day. Western blots of RAGE, c-Fos, and NFATc1 are shown. All data are presented as the mean ± SD

The S100 family of proteins has been shown to bind to the RAGE and Toll-like receptor 4 (TLR4) receptors to mediate tumor growth and survival.^18,19^ The cell surface protein CD44 has also been implicated in S100A4-induced cytoskeletal changes in melanoma.^20^ Therefore, we explored whether S100A4 utilizes one of these surface receptors for osteoclastogenesis. Osteoclast formation from pre-osteoclasts with reduced levels of RAGE, CD44, or TLR4 was compared with that from control cells after culturing in the presence of rS100A4. When a substantial reduction in RAGE expression was achieved by transfecting small interfering RNA oligonucleotides (Supplementary Fig. 4a, b), osteoclast formation was significantly decreased (Fig. 5a). In contrast, CD44 knockdown (Supplementary Fig. 5a, b) and TLR4 knockout (Supplementary Fig. 5c, d) did not have significant effects. Consistently, S100A4 induction of osteoclast marker gene expression was reduced by RAGE knockdown (Supplementary Fig. 4c). In addition, RAGE knockdown led to decreased levels of osteoclast formation and bone resorption in mtMDA CM-treated cultures (Fig. 5b and Supplementary Fig. 6). Similarly, mtMDA-Csh-CM-induced osteoclastogenesis was reduced by RAGE knockdown (Fig. 5c). In contrast, osteoclastogenesis with mtMDA-S100A4sh CM was not significantly different between the RAGE knockdown and control knockdown groups (Fig. 5c). In line with these results, the induction of c-Fos and NFATc1 by mtMDA CM or rS100A4 was attenuated by RAGE knockdown (Fig. 5d).

**Fig. 6:**
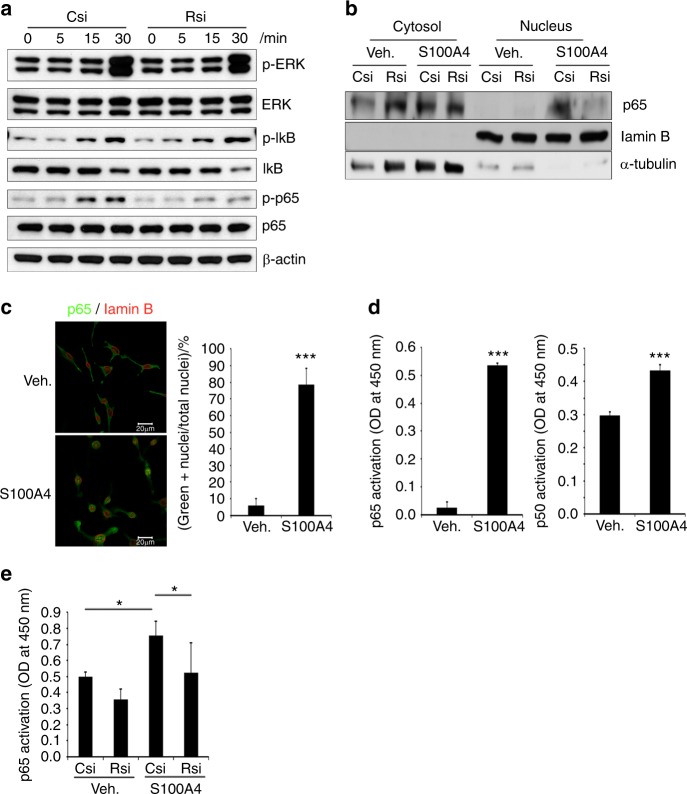
S100A4 activates canonical nuclear factor (NF)-κB via RAGE (receptor for advanced glycation end products). **a** Csi- or Rsi-transfected cells were stimulated with rS100A4 (2 μg·mL^−1^) for the indicated times. Whole-cell lysates were subjected to western blotting analysis. **b** Nuclear and cytosolic fractions were prepared from Csi- or Rsi-transfected cells treated with Veh. or rS100A4 (2 μg·mL^−1^) for 1 h and subjected to western blot analysis. **c** Confocal microscopy of pre-osteoclasts (pre-OCs) stimulated with rS100A4 (2 μg·mL^−1^) for 1 h after staining with p65 and lamin B Abs. Cells with nuclear p65 staining were counted. *n* = 3 per group. ***P < 0.001 by unpaired two-tailed Student’s *t* test. Scale bars, 20 μm. **d** Transcription factor activity assays for p65 and p50 NF-κB with nuclear fractions from pre-OCs stimulated with S100A4 for 1 h. *n* = 3 (left graph) or 4 (right graph) per group. ***P < 0.001 by unpaired two-tailed Student’s *t* test. **e** Nuclear fractions were subjected to the p65 transcription factor assay. *n* = 5 per group. *P < 0.05 by two-way analysis of variance with post hoc Tukey’s test

To gain further insights into the mechanism involved in the stimulation of osteoclastogenesis by S100A4 from mtMDA, we next examined the intracellular signaling pathways reported to be activated during osteoclast differentiation. Recombinant S100A4 stimulated the phosphorylation of mitogen-activated protein kinases, IκB, and p65 NF-κB in pre-osteoclasts (Supplementary Fig. 7). While the phosphorylation of ERK and IκB stimulated by S100A4 was not affected by RAGE knockdown, the phosphorylation of p65 was clearly diminished by RAGE knockdown (Fig. 6a). In addition, the S100A4-dependent increase in p65 in nuclear fractions was inhibited in RAGE knockdown cells (Fig. 6b). Confocal microscopy also showed that S100A4 increased the percentage of cells with nuclear p65 (Fig. 6c). Furthermore, S100A4 stimulated the transcriptional activity of canonical NF-κB p65 and, to a lesser extent, p50 (Fig. 6d). The noncanonical NF-κB, RelB, and p52 were not activated by S100A4 (Supplementary Fig. 8). RAGE knockdown negated the effect of S100A4 on p65 NF-κB activity (Fig. 6e). Collectively, these results demonstrated that RAGE plays a pivotal role in S100A4 secretion from mtMDA to enhance osteoclastogenesis by stimulating p65 NF-κB.

### S100A4 is responsible for bone loss caused by metastatic breast cancer cells

**Fig. 7:**
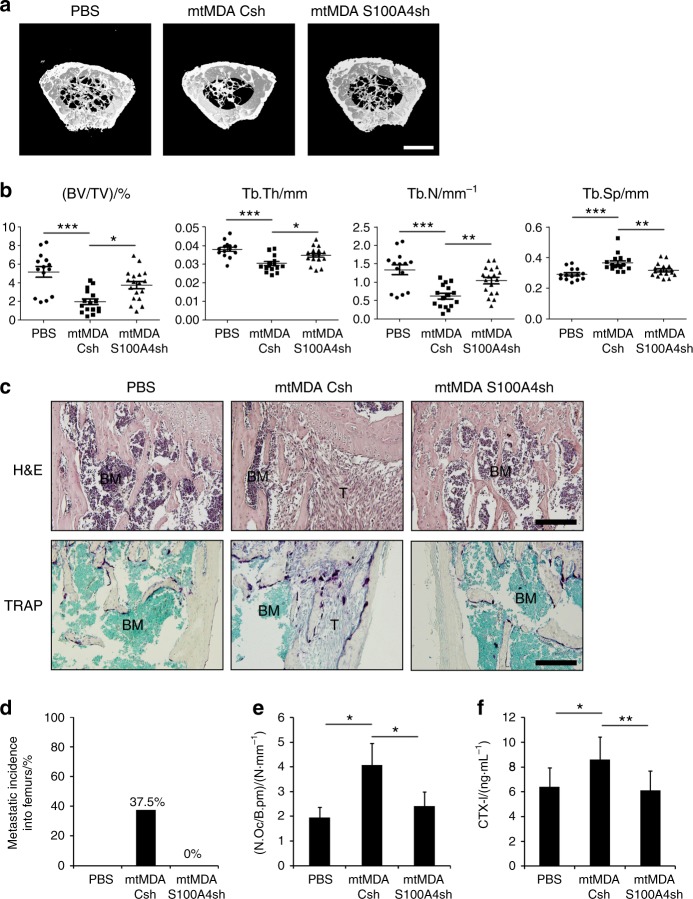
S100A4 mediates bone metastasis and osteolysis by mtMDA in vivo. Microcomputed tomographic (μCT) analyses of femurs from BALB/c-nude mice that received intracardiac injection of phosphate-buffered saline (PBS), mtMDA-Csh cells, or mtMDA-S100A4sh cells. *n* = 14 from 7 mice for PBS, *n* = 16 from 8 mice for mtMDA-Csh, and *n* = 18 from 9 mice for mtMDA-S100A4sh. **a** Representative three-dimensional-reconstructed μCT images. Scale bars, 0.7 mm. **b** Quantitative analyses of μCT data show bone volume per tissue volume (BV/TV), trabecular thickness (Tb.Th), trabecular number (Tb.N), and trabecular separation (Tb.Sp). *P < 0.05, **P < 0.01, ***P < 0.001 by one-way analysis of variance (ANOVA) with post hoc Tukey’s test. **c** Hematoxylin & eosin (H&E)-stained (upper) and tartrate-resistant acid phosphatase (TRAP)-stained (bottom) images of decalcified femur sections from **a**. Scale bars, 200 μm. BM bone marrow, T tumor. **d** Metastatic incidence was measured with H&E-stained sections. **e** Number of osteoclasts per bone perimeter (N.Oc/B.pm) was analyzed with TRAP-stained sections. *P < 0.05 by one-way ANOVA with post hoc Tukey’s test. **f** Serum CTX-I levels were measured by enzyme-linked immunosorbent assay. *P < 0.05, **P < 0.01 by one-way ANOVA with post hoc Tukey’s test. All data are presented as the mean ± SD

The elevated secretion of S100A4 from bone-metastatic mtMDA cells (Fig. 2) and the stimulation of osteoclastogenesis by S100A4 (Fig. 4) led us to next investigate the in vivo relevance of S100A4 in the bone destruction induced by bone-metastasized cancers. We performed radiographic and histologic analyses of femurs from female nude mice that had received intracardiac injection of mtMDA-Csh or mtMDA-S100A4sh cells. μCT scans showed that the bone mass was decreased by injection of cancer cells and that the extent of bone mass reduction was much weaker in mtMDA-S100A4sh-injected mice than in mtMDA-Csh-injected mice (Fig. 7a). Quantitative analyses of trabecular bone revealed significantly higher bone volume per tissue volume (BV/TV), trabecular thickness (Tb.Th), and trabecular number (Tb.N) in mtMDA-S100A4sh-injected mice than in mtMDA-Csh-injected mice (Fig. 7b). Trabecular separation (Tb.Sp) was significantly lower in mtMDA-S100A4sh-injected mice than in mtMDA-Csh-injected mice. Hematoxylin and eosin (H&E) staining of decalcified bone tissue sections showed that the metastatic incidence of tumors was much higher in mtMDA-Csh-injected mice than in mtMDA-S100A4sh-injected mice (Fig. 7c, d). In TRAP staining, the number of osteoclasts per bone perimeter (N.Oc/B.Pm) was significantly higher in mtMDA-Csh-injected mice than in mtMDA-S100A4sh-injected mice (Fig. 7c, e). Consistently, serum CTX-I levels were lower in mtMDA-S100A4sh-injected mice than in mtMDA-Csh-injected mice (Fig. 7f).

**Fig. 8:**
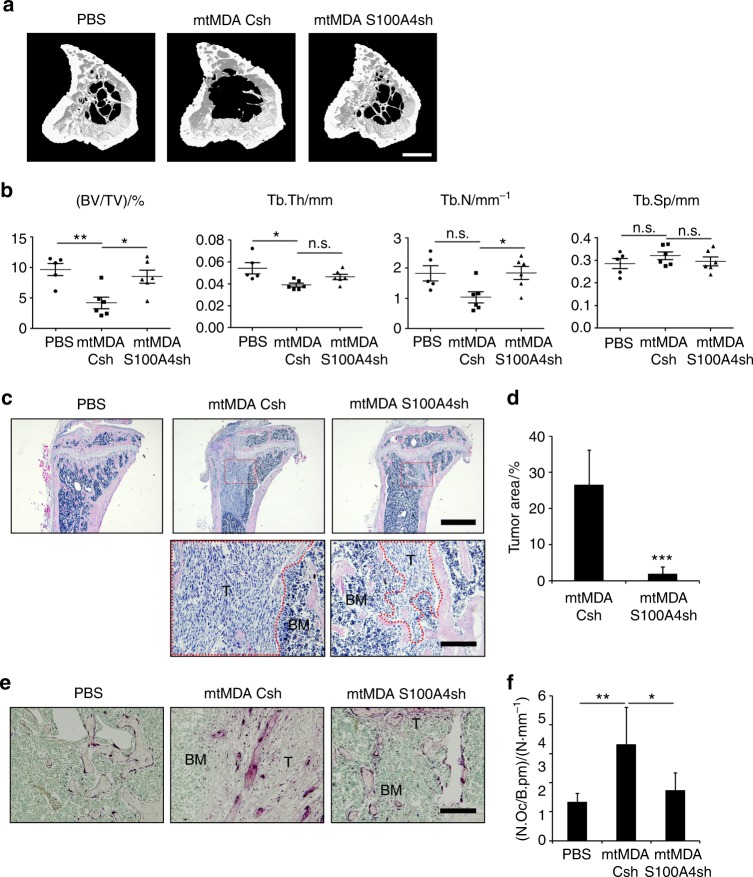
In vivo bone destruction is stimulated by S100A4 from mtMDA in a direct cancer inoculation model. Microcomputed tomographic (μCT) analyses of tibias from BALB/c-nude mice that received intratibial injection of phosphate-buffered saline (PBS), mtMDA-Csh, or mtMDA-S100A4sh cells. *n* = 5 for PBS, *n* = 6 for mtMDA-Csh, and *n* = 6 for mtMDA-S100A4sh. **a** Representative three-dimensional-reconstructed μCT images of tibias. Scale bars, 0.5 mm. **b** Quantitative analyses of μCT data show bone volume per tissue volume (BV/TV), trabecular thickness (Tb.Th), trabecular number (Tb.N), and trabecular separation (Tb.Sp). *P < 0.05, **P < 0.01, by one-way analysis of variance (ANOVA) with post hoc Tukey’s test. n.s. not significant. **c** Hematoxylin & eosin (H&E)-stained images of decalcified tibia sections from **a**. Scale bars, 1 mm (upper) and 200 μm (bottom). BM bone marrow, T tumor. **d** Tumor areas were measured with H&E-stained sections. ***P < 0.001 by unpaired two-tailed Student’s *t* test. **e** Tartrate-resistant acid phosphatase (TRAP)-stained images of decalcified tibia sections. Scale bars, 200 μm. BM bone marrow, T tumor. **f** Number of osteoclasts per bone perimeter (N.Oc/B.pm) was analyzed with TRAP-stained sections. *P < 0.05, **P < 0.01 by one-way ANOVA with post hoc Tukey’s test. All data are presented as the mean ± SD

As the histological analysis of femurs from mice that received intracardiac injection of mtMDA-S100A4sh revealed hardly any tumor mass in bones (Fig. 7d), we next sought to more directly assess the in vivo effect of S100A4 from mtMDA cells on osteoclasts. To this end, we directly injected mtMDA-Csh or mtMDA-S100A4sh cells into the tibia of female nude mice. Tumor cell-induced bone destruction was significantly higher in mtMDA-Csh-injected mice than in mtMDA-S100A4sh-injected mice (Fig. 8a, b). The tumor area was much higher in mtMDA-Csh-injected mice than in mtMDA-S100A4sh-injected mice (Fig. 8c, d). In addition, a higher number of osteoclasts was detected in mtMDA-Csh-injected tibia than in mtMDA-S100A4sh-injected tibia (Fig. 8e, f). Taken together, these data suggest that S100A4 released by bone metastatic MDA cells plays critical roles in stimulating osteolysis and increasing tumor burden in the bone marrow.

### S100A4 blockade ameliorates bone destruction by breast cancer metastasis

**Fig. 9:**
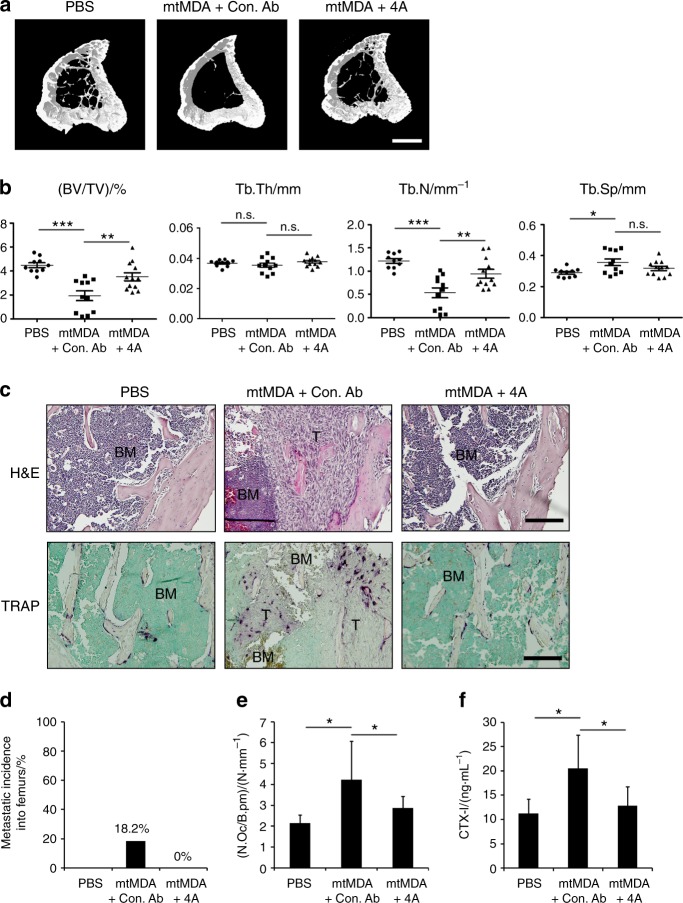
S100A4-blocking antibody (Ab) reduces bone metastasis of breast cancer cells. Microcomputed tomographic (μCT) analyses of tibias from BALB/c-nude mice that received intraperitoneal injection of control monoclonal Ab (mAb; Con. Ab) or S100A4 mAb (4A) after intracardiac injection of mtMDA. *n* = 10 from 5 mice for phosphate-buffered saline, *n* = 12 from 6 mice for Con. Ab, and *n* = 12 from 6 mice for 4 A. **a** Representative three-dimensional-reconstructed μCT images of tibias. Scale bars, 0.5 mm. **b** Quantitative analyses of μCT data show bone volume per tissue volume (BV/TV), trabecular thickness (Tb.Th), trabecular number (Tb.N), and trabecular separation (Tb.Sp). *P < 0.05, **P < 0.01, ***P < 0.001 by one-way analysis of variance (ANOVA) with post hoc Tukey’s test. n.s. not significant. **c** Hematoxylin & eosin (H&E)-stained images (upper) and tartrate-resistant acid phosphatase (TRAP)-stained images (bottom) of decalcified tibia sections from a. Scale bars, 200 μm. BM bone marrow, T tumor. **d** Metastatic incidence was measured with H&E-stained sections. **e** Number of osteoclasts per bone perimeter (N.Oc/B.pm) was analyzed with TRAP-stained sections. *P < 0.05 by one-way ANOVA with post hoc Tukey’s test. **f** Serum CTX-I levels were measured by enzyme-linked immunosorbent assays. *P < 0.05, by one-way ANOVA with post hoc Tukey’s test. All data are presented as the mean ± SD

We next investigated whether systemic administration of a S100A4-blocking antibody would have beneficial effects on bone metastasis of mtMDA cells. To this end, we raised a monoclonal Ab (mAb) against human S100A4 using phage display technology. One of the mAb clones (4A) effectively reduced in vitro osteoclast differentiation in the culture with mtMDA CM (Supplementary Fig. 9). The 4A mAb or a negative control mAb was intraperitoneally administered to nude mice that had also received intracardiac injection of mtMDA. Tibiae of 4A-treated mice showed significantly higher BV/TV and Tb.N values in μCT analysis than those of the controls (Fig. 9a, b). In the analyses of stained sections, the metastatic incidence of mtMDA was completely blocked by 4A (Fig. 9c, d). N.OC/B.Pm was lower in TRAP-stained sections from 4A-treated mice than in those from control Ab-treated mice (Fig. 9c, e). In addition, serum CTX-I levels were lower in 4A-treated mice than in control mice (Fig. 9f). These results indicate that blocking S100A4 was effective in suppressing skeletal destruction by bone metastasis of breast cancer cells.

## Discussion

In this study, we found that S100A4 is secreted from bone-metastatic breast cancer cells. This secretion seems to be responsible for the stimulation of osteoclastogenesis by mtMDA because adding neutralizing Ab to the CM or shRNA-mediated gene knockdown in cancer cells nullified the stimulatory effect. In corroboration, treatment with rS100A4 protein increased osteoclast formation and bone resorption both in vitro and in vivo. Furthermore, in vivo injection of mtMDA-S100A4sh cells led to less osteolysis than injection of mtMDA-Csh. Moreover, a S100A4-blocking Ab attenuated mtMDA-induced bone loss in vivo. Therefore, we propose that S100A4 is a major contributor to osteoclast development and bone destruction associated with breast cancer bone metastasis.

The communication between breast cancer cells and skeletal cells in bone metastasis is mediated by multiple components, including factors released from tumor cells. Among the tumor-derived factors, PTHrP is the most extensively studied. The role of PTHrP in bone metastasis is multifaceted; the autocrine action of PTHrP promotes the proliferation, survival, invasion, and migration of tumor cells, while the paracrine and endocrine action of PTHrP modulates bone-metastatic environments.^21^ The primary mechanism by which PTHrP modulates the bone microenvironment seems to be indirect stimulation of osteoclastogenesis via induction of RANKL in osteoblasts and osteocytes.^21^ This indirect mode of action appears to be distinct from the direct stimulation of osteoclastogenesis by S100A4 in our present study. However, S100A4 may have a dual function: directly acting on pre-osteoclasts via RAGE and indirectly functioning via RANKL induction in osteoblasts. In support of this notion, we observed upregulation of RANKL by S100A4 in mouse calvarial osteoblasts (data not shown). This type of dual action has also been reported with interleukin 8, levels of which correlate with increased bone metastasis.^22^ In the vicious cycle model of bone metastasis, PTHrP secretion from tumor cells is further stimulated by bone-latent factors, such as TGF-β, released upon matrix resorption by osteoclasts.^2,23^ Whether S100A4 stimulation of osteoclasts would lead to further induction of S100A4 in tumor cells, forming another type of vicious cycle in bone-metastatic environments, is a question to be resolved.

Recently, modified views of our understanding of tumor bone metastasis have added dormancy and reactivation stages prior to the vicious cycle stage between tumor cells and osteoclasts.^5^ Because osteoclasts may also have a role in the reactivation process, it would be intriguing to investigate the role of S100A4 in the regulation of dormancy induction or reactivation of breast cancer cells in the bone-metastatic niche. The interplay between tumor cells and stroma was suggested to enhance S100A4 secretion, which leads to modulation of the tumor microenvironment via stimulation of angiogenesis and MMP activity.^24^ In addition, infiltration of immune cells, including macrophages and T cells, is an important factor in the metastatic niche, and S100A4 was shown to be crucial to the recruitment of T cells.^25–27^ In support of the role of S100A4 in modulating the niche where immune cells participate, a S100A4-neutralizing Ab was shown to suppress T cell recruitment and premetastatic niche formation, inhibiting lung metastasis of mouse mammary carcinoma cells.^28^ Because macrophages can serve as precursors of osteoclasts, increased S100A4 levels in the bone-metastatic niche may recruit osteoclast precursors on one hand and stimulate osteoclast differentiation, as demonstrated in our study, on the other hand, in bone metastasis. Tumor-released S100A4 may stimulate stromal components such as osteoblast-lineage cells to mediate their interplay with osteoclasts, driving the microenvironment favorable for reactivation and growth of breast cancer cells in the bone-metastatic niche.

In addition to promoting tumor growth and dissemination in various types of murine and human cancers,^8^ S100A4 was shown to stimulate invasion and migration by adjusting the extracellular matrix, cell membrane fluidity, and the cytoskeleton of murine melanoma cells.^29^ When we compared cell proliferation, migration, and invasion between mtMDA and parental MDA cells, mtMDA cells displayed greater migration and invasion than did MDA cells with no difference in proliferation (data not shown). In addition, S100A4 knockdown in mtMDA cells decreased migration or invasion in vitro (data not shown). These effects on migration and invasion may be derived from the intracellular function of S100A4 or the autocrine action of secreted S100A4. Under in vivo conditions, secreted S100A4 may also influence neighboring cells and matrix in a paracrine way during the process of dissemination and establishment in a distant site. Our in vivo model of intracardiac injection of tumor cells showed that the tumor burden was decreased in mice that received mtMDA-S100A4sh compared to mice injected with mtMDA-Csh (Fig. 7). This effect on bone metastatic incidence may be in part attributed to cell-intrinsic differences. However, injection of the cancer cells directly into bone also resulted in a lower cancer mass in mtMDA-S100A4sh-injected mice than in mtMDA-Csh-injected mice (Fig. 8), suggesting that cancer-derived S100A4 modulates the bone microenvironment to support cancer cell growth. The function of S100A4 in the metastasis of breast cancer is not limited to bone. S100A4 expression was also increased in breast cancer that metastasized to lung, brain, and liver (Fig. 2b). Moreover, several studies have reported that S100A4 regulates breast cancer cell migration to other organs in animal models. In a study by Wang et al., knockdown of S100A4 was shown to inhibit lung metastasis of MDA-MB-231 cells,^30^ and a study by Liu et al. reported that recombinant S100A4 promoted migration of 4T1 mouse breast cancer cells to the lung, liver, and kidney.^31^ Therefore, S100A4 is an attractive therapeutic target for the metastasis of breast cancers to multiple organs, including bone.

S100A4 has been shown to utilize cell surface receptors, including RAGE, TLR4, CD44, and annexin 2, in diverse cell types.^32^ In particular, RAGE has been implicated in the metastasis of several types of cancers, including lung, brain, and prostate cancer and osteosarcoma.^33–35^ Here we demonstrated that RAGE served as the receptor for S100A4 in stimulating osteoclastogenesis. Intriguingly, RAGE knockout mice displayed an osteopetrotic phenotype due to defects in osteoclast maturation and function.^36^ High-mobility group box 1 (HMGB1) was suggested as a ligand of RAGE in support of osteoclastogenesis.^37^ In addition, the HMGB1–RAGE interaction has been shown to have significant roles in the metastasis of lung and brain cancers.^33^ However, our microarray analysis showed that HMGB1 expression was lower in MDA cells than in MCF7 cells, suggesting that HMGB1 is unlikely to contribute to the osteoclastogenesis occurring in breast cancer metastasis. In conclusion, targeting the S100A4–RAGE interaction is a valid approach for inhibiting the bone destruction caused by breast cancer metastasis.

In our previous study, we found that rS100A4 treatment inhibited the matrix mineralizing activity of osteoblasts via the NF-κB signaling pathway.^38^ Consequently, S100A4 from bone metastases will drive highly catabolic bone metabolism as a result of impaired osteoblast function and enhanced osteoclast activity. Owing to the dual action of S100A4 on osteoblasts and osteoclasts, measures to block S100A4 function, such as the mAb developed in our study, could lead to effective bone mass accrual. Thus targeting S100A4 may be ideal in conditions of severe bone loss such as metastasis-induced osteolysis. In addition, S100A4 does not seem to play a critical role in normal physiology. Its expression is restricted in normal adult tissues, and S100A4 knockout mice displayed no evident abnormalities.^39^ In contrast, S100A4 expression is highly upregulated in disease states such as tissue fibrosis, rheumatoid arthritis, and cancer. These aspects make S100A4 an attractive molecular target for the development of therapeutics with minimal adverse effects. To our knowledge, this study is the first to demonstrate the role of tumor-derived S100A4 in metastasis-associated osteoclastogenesis and the first to project the concept of blocking S100A4 as an effective strategy in the treatment of cancer-induced bone destruction.

## Materials and Methods

### Establishment of the mtMDA cell line

For establishment of a highly bone-metastatic mtMDA cell line, in vivo selection was performed as previously described.^17^ MDA-MB-231 cells (MDA; 1 × 10^5^) in 100 μL phosphate-buffered saline (PBS) were injected into the left ventricle of 9-week-old female BALB/c-nude mice using a 1-mL syringe with a 26-G needle. After 12 weeks, the bone marrow of femurs and tibiae were flushed, and the collected cells expanded in culture for 8 weeks. Cultured cells were reinjected into the left ventricle for another round of selection. Cells were harvested at 8 weeks after injection and cultured for 8 weeks. Selected cancer cells were named mtMDA.

### Mouse experiments

Animal experiments were performed in compliance with ethical regulations and approved by the Committees on the Care and Use of Animals in Research at Seoul National University. Mice were kept at a specific pathogen-free facility of Seoul National University. For in vivo rS100A4 injection experiments, 6-week-old female ICR mice were randomly assigned to vehicle and S100A4 groups (5 mice per group). Mice were subcutaneously injected in the calvariae with 10 μg of rS100A4 diluted in PBS (50 μL/injection) or an equal volume of vehicle diluted in PBS every other day for 8 days. For mtMDA xenograft model experiments, 1 × 10^6^ cells in 100 μL PBS were injected into the left ventricle of 6-week-old female BALB/c-nude mice. Mice were randomly assigned to the PBS (seven mice), mtMDA-Csh (eight mice), and mtMDA-S100A4sh (nine mice) groups. After 4 weeks, femurs from both legs were subjected to μCT or to histology analysis after decalcification. In intratibial tumor experiments, 6-week-old female BALB/c-nude mice were randomly assigned to the PBS (five mice), mtMDA-Csh (six mice), and mtMDA-S100A4sh (six mice) groups. PBS (5 μL) or mtMDA cells (5 × 10^5^) were directly injected into the left tibias of mice. Mice were sacrificed at 3 weeks after mtMDA injection, and the left tibias were collected for analysis. For in vivo neutralizing Ab experiments, PBS (5 mice) or mtMDA cells (1 × 10^6^; 12 mice) were injected into the left ventricle of 6-week-old female BALB/c-nude mice. Then mtMDA-injected mice were randomly assigned into the control antibody (anti-human respiratory syncytial virus Ab, six mice) and anti-S100A4 neutralizing antibody (4A Ab, six mice) groups. Starting from 1 day before mtMDA injection, S100A4 neutralizing 4A Ab or the control Ab (5 mg·kg^–1^ per injection) was given intraperitoneally twice weekly. Mice were sacrificed at 4 weeks after mtMDA injection, and tibias from both legs were analyzed.

### CM collection

A total of 1 × 10^6^ cancer cells were seeded onto 60-mm tissue culture plates with Dulbecco’s modified Eagle’s medium supplemented with 10% fetal bovine serum (FBS). The next day, the medium was changed to alpha-Minimum Essential Medium with 10% FBS and incubated for 24 h. The medium was collected and centrifuged for 5 min at 1 200 r·min^–1^. The supernatant (CM) was stored at −80 °C for future use. A portion (30%) of CM (70% of fresh medium) was used unless otherwise specifically indicated.

### Osteoclast culture

BMMs were prepared by using 5-week-old female ICR mice as previously described.^40^ BMMs were committed to the pre-osteoclast lineage by treating them with RANKL (50 ng·mL^–1^) and macrophages colony-stimulating factor (M-CSF; 30 ng·mL^–1^) for 36 h–48 h before adding CM from cancer cells. Pre-osteoclasts were incubated with M-CSF (30 ng·mL^–1^) and cancer cell CM. TRAP^+^ multinucleated mature osteoclasts were usually observed at 24 h–48 h after treatment with CM. TRAP staining was carried out with a Leukocyte Acid Phosphatase Kit (Sigma Aldrich). TRAP^+^ cells with more than three nuclei were counted as osteoclasts by using an Olympus BX51 microscope equipped with the DP2-BSW software (version 2.2).

### Microcomputed tomography

For the experiment shown in Fig. 1b, femurs were analyzed with a 1 072 Microtomograph (SkyScan). A total of 320–350 tomographic slices at a 4-μm resolution were acquired. We performed three-dimensional analyses with the V-Works program (Cybermed). For the animal experiments except the one shown in Fig. 1b, mouse calvariae, femurs, and tibias were analyzed with a SkyScan 1 172 scanner (40 kV, 250 μA, 15 μm pixel size for calvariae; 70 kV, 142 μA, 7 μm pixel size for femurs and tibias). Calvarial bone volumes were assessed by analyzing 5-mm regions between the occipital and the frontal calvarial bones with the CT-analyzer program (threshold 95–255, version 1.7, SkyScan). Trabecular bone volumes of femurs and tibias were assessed by analyzing 0.5-mm regions below the growth plate (threshold 120–255, version 1.7, SkyScan). Three-dimensional images were obtained by using the CT-volume program (version 1.11, SkyScan).

### Histology

Paraffin-embedded sections of decalcified bones were prepared as described previously.^41^ Calvariae, femurs, and tibias were fixed with 4% paraformaldehyde overnight and decalcified in 12% EDTA solution for 4–5 weeks. Decalcified samples were embedded in paraffin and sliced into 6-μm-thick sections with a Leica microtome RM2145 (Leica Microsystems). Sliced specimens were baked for 30 min and soaked in xylene for paraffin removal. After a series of hydration steps, the sections were subjected to H&E staining or TRAP staining followed by methyl green counterstaining. Two to three sections per mouse from equivalent regions of bones were analyzed for Oc.S/BS by using the Osteomeasure program (version 2.2.0.3, OsteoMetrics, Inc.).

### S100A4 neutralizing mAb generation

A phage display of chicken single-chain variable fragment (scFv) libraries was constructed as previously described^42^ after immunization of leghorn chickens with human rS100A4 protein. Four rounds of biopanning were performed with magnetic beads (Dynabeads M-270 epoxy, Invitrogen) coated with human rS100A4. The scFv-displaying phages were subjected to phage enzyme immunoassays, as described previously.^43^ Plasmid DNA was prepared from selected clones that had binding activity for human rS100A4. The sequence of the V_H_ and V_L_ chains of the scFv Ab was identified. The gene encoding scFv was fused to human Fc and subcloned into a modified pCEP4 mammalian expression vector. mAbs were produced by transient transfection of HEK293F cells (Invitrogen) using polyethylenimine (Polysciences) as described previously.^44^ After 5 days, supernatants were collected, and mAbs were purified by protein A gel chromatography (Repligen) according to the manufacturer’s instructions. Purified Abs were desalted against PBS by using a desalting column (Thermo Fisher Scientific).

### Statistical analysis

Data are presented as the mean ± SD of biological replicates. All experiments, except in vivo mouse studies, were performed at least three times. The sample size chosen in this study was similar to those generally employed in the field.^45^ None of the samples or animals were excluded from the analysis. Although investigators were not blinded, animals for each experiment were randomly selected for further treatments. An unpaired two-tailed Student’s *t* test was used to assess differences between two groups. One-way or two-way analysis of variance with post hoc Bonferroni or Tukey’s test was used for analyses of multiple groups. All statistical tests were performed using SigmaPlot 11.0 (Version 11.2.0.11, Systat software Inc., San Jose, CA, USA). Statistical tests were justified as appropriate for every figure. Statistical significance was considered after determination of the normality and the equal variance test. A *P* value <0.05 was considered significant.

## Supplementary information

Supplementary Information

## Supplementary information

**Supplementary information:** The online version of this article (10.1038/s41413-019-0068-5) contains supplementary material, which is available to authorized users.
