# Supplementary material for: S100A4 released from highly bone-metastatic breast cancer cells plays a critical role in osteolysis
Source: Bone Res. 2019 Sep 23;7:30. doi: 10.1038/s41413-019-0068-5 (PMC6804941; doi:10.1038/s41413-019-0068-5)
Supplement: Supplementary file 1 — Supplementary Information [file 41413_2019_68_MOESM1_ESM.docx]

**SUPPLEMENTARY INFORMATION**

**SUPPLEMENTARY MATERIALS AND METHODS**

Cell Lines and Culture Conditions

MDA-MB-231 (MDA) and MCF7 cells were purchased from the Korean Cell Line Bank (Seoul, Korea). Cell lines were validated by STR-PCR analysis by the Korean Cell Line Bank and the STR profiles of each cell line can be accessed from the website of the Korean Cell Line Bank (https://cellbank.snu.ac.kr/main/tmpl/sub_main.php?m_cd=22&m_id=0503). Mycoplasma contamination tests were also performed by the Korean Cell Line Bank. Cells were maintained in high-glucose DMEM (Lonza) supplemented with 10% FBS (Life Technologies) and 1% penicillin and streptomycin (WELGENE, Korea).

Reagents

Recombinant mouse S100A4 was purchased from Prospec. Recombinant M-CSF and RANKL were from PeproTech. Recombinant OPG was purchased from R&D Systems. Antibody against c-Fos (sc-7202) was purchased from Santa Cruz. Phosphospecific antibodies against ERK (Thr^202^/Tyr^204^) (#9101), JNK (Thr^182^/Tyr^185^) (#9251), p38 (Thr^180^/Tyr^182^) (#9211), and I*k*B (Ser^32^) (#2859), and antibodies against p65 (Ser^536^) (#3031), ERK (#9102), JNK (#9252), p38 (#9212), I*κ*B (#9242), and p65 (#8242) were obtained from Cell Signaling Technology. Antibodies for RAGE (DD/A11) (#MAB5328) were from Millipore. Anti-S100A4 (ab27957) used in Fig. 4F was purchased from Abcam. Antibodies for NFATc1 (7A6) (#556602) and hHLA (#551337) were purchased from BD Pharmingen. Anti-β-actin antibody (AC-74) (A2228) and the leukocyte acid phosphatase kit (for TRAP staining) were purchased from Sigma-Aldrich. Dentin slices were purchased from Immunodiagnostic Systems.

Flow Cytometry

Human cancer cells were validated by labelling with PE-conjugated mouse anti-human β2-microglobulin, which is associated with the HLA Class I antigen complex. Flow cytometry analyses were performed utilizing human embryonic kidney-293 cells as a positive control and mouse bone marrow cells as a negative control. Collected cells were resuspended in ice-cold PBS at the concentration of 1 x 10^5^ cells in 100 μL PBS. Then, cells were incubated with the antibody (1:100) for 30 min in the dark and washed three times with ice-cold PBS. Cells were resuspended in ice-cold PBS and immediately subjected to flow cytometry analysis with FACSCalibur (BD Science).

Western Blotting

Cells were lysed with RIPA buffer (10 mM Tris pH 7.2, 150 mM NaCl, 0.1% sodium dodecyl sulfate, 1% Triton X-100, 1% sodium deoxycholate, 5 mM ethylenediaminetetraacetic acid) after washing with cold PBS. Western blotting was performed with thirty micrograms of cell lysates following a standard protocol. For Western blotting experiments with nuclear and cytoplasmic fractions, fractions of cells were prepared by using the Nuclear Extract Kit from Active Motif (Carlsbad) following the manufacturer’s instruction. Twenty micrograms of nuclear fraction and thirty micrograms of cytoplasmic fraction were used for Western blotting analyses. As loading controls for cytosolic and nuclear fractions, alpha-tubulin and lamin B were used, respectively.

Real-time PCR Analysis

The quantification of mRNA levels by real-time PCR analysis was performed following a standard protocol. Primers for real-time PCR analyses are as follows: *hprt* (hypoxanthine-guanine phosphoribosyltransferase) forward, 5’-cctaagatgagcgcaagttgaa-3’; *hprt* reverse, 5’- ccacagggactagaacacctgctaa-3’; *atp6v0d2* forward, 5’-gggagaccctcttccccacc-3’; *atp6v0d2* reverse, 5’-ccaccgacagcgtcaaacaaa-3’; *mmp2* forward, 5'-cactgaccagcctggggttg-3'; *mmp2* reverse, 5’-agggaaaccggggtccattt-3’; *mmp9* forward, 5'-gacggcacgccttggtgtag-3'; *mmp9* reverse, 5’-aggagcggccctcaaagatg-3’; *oscar* forward, 5'-gctgacttcacaccaacagc-3'; *oscar* reverse, 5’- gggtgacaaggccacttt-3’; *acp5* forward, 5'-cgaccattgttagccacatacg-3'; *acp5* reverse, 5’- tcgtcctgaagatactgcaggtt-3’; *c-Fos* forward, 5'- acttcttgtttccggc-3'; *c-Fos* reverse, 5’-agcttcagggtaggtg-3’; *NFATc1* forward, 5'- ccagtataccagctctgcca-3'; *NFATc1* reverse, 5’-gtgggaagtcagaagtgggt-3’; *ctsk* forward, 5'-atatgtgggccaccatgaaagtt-3'; *ctsk* reverse, 5’-tcgttccccacaggaatctct-3’; *dcstamp* forward, 5'-gggtgctgtttgccgctg-3'; *dcstamp* reverse, 5’-cgactccttgggttccttgct-3’; human *hprt* forward, 5’- accccacgaagtgttggata-3’; human *hprt* reverse, 5’- aagcagatggccacagaact-3’; human *s100a4* forward, 5’-gcccagcttcttggggaaaa-3’; human *s100a4* reverse, 5’- atggcgatgcaggacaggaa-3’

RNA Interference

In the RNA interference experiments, lentiviral particles containing shRNA targeting human S100A4 and siRNA oligonucleotides targeting mouse RAGE were used. Lentiviral particles containing shRNA were purchased from Santa Cruz (Control shRNA, #sc-108080; S100A4 shRNA, #sc-149694-V). S100A4-targetted lentiviral particles contain two different sequences (A: 5’–GATCCCATGATGTGCAATGAATTCTTCAAGAGAGAATTCATTGCACATCATGTTTTT–3’, B: 5’- GATCCGCTGTGAGATAGTGCCTTATTCAAGAGATAAGGCACTATCTCAC

AGCTTTTT-3’). Cells were infected with lentiviruses at a multiplicity of infection of 100 for 24 hours in complete medium in the presence of polybrene (8 μg/mL; Sigma Aldrich). Lentivirus-infected cells were cultured for 7 days with puromycin (2 μg/mL) to remove uninfected cells. For RAGE knockdown experiments, a control siRNA (#SC-37007) and RAGE siRNA (#sc-36375) were purchased from Santa Cruz. RAGE siRNA contains pool of three different siRNA sequences (A: 5’-GAACACAGGAAGAACUGAAtt-3’, B: 5’-GAUUCCCGAUGGCAAAGAAtt-3’, C: 5’-GCAUUCAGCUGUUGGUUGAtt-3’). Cells were transfected with a complex formed by mixing 40 nM of siRNA and 9 μL of HiPerFect (QIAGEN, Montreal, CA) dissolved in 100 μL of Opti-MEM (Life Technologies, Gaithersburg, MD) for 6 hours. After 48 hours of infection or transfection, cells were analyzed for the silencing efficiency by measuring mRNA levels by real-time PCR and protein levels by Western blotting.

Immunofluorescence Microscopy

Pre-OCs were serum-starved for 5 hours and stimulated with mouse rS100A4 (2 μg/mL) for 1 hour. Cells were fixed with 3.7% formaldehyde, and permeabilized with 0.1% Triton X-100 for 15 minutes. After blocking nonspecific sites with 1% bovine serum albumin (BSA)-PBS for 2 hours, anti-p65 (1/100 dilution) was added. After overnight incubation, cells were incubated with anti-lamin B (1/50 dilution) for 2 hours, followed by incubation with secondary rabbit-FITC and goat-Cy3 (1/300 dilution) Abs for 1 hour. Nuclei were stained by 4',6-diamidino-2-phenylindole (DAPI) mounting medium (Vectashield, Vector Laboratories). The prepared slides were viewed under a Zeiss LSM 700 laser-scanning microscope (Carl Zeiss Microimaging GmbH). Objective lenses: C-Apochromat 40x / 1.20 W. Detectors: PMT. Filters: DAPI (BP 420-475), Green (BP 490-555), and Red (560 IF). Lasers: 405 nm, 488 nm, and 555 nm. Pictures were taken and analyzed with the ZEN2010 program (version 6.0.0.320).

Enzyme-linked Immunosorbent Assay (ELISA)

Cancer cells were seeded at 1 x 10^5^ cells/well into a 48-well tissue culture plate with DMEM supplemented with 10% FBS. After overnight incubation, media were replaced with 100 μL serum-free media and cells were further cultured for 24 hours. Culture supernatant was collected and subjected to ELISA with a human S100A4 ELISA kit (CycLex Co.) following the manufacturer’s instruction.

Transcription Factor Activity Assay

Nuclear fractions of cells were prepared by using the Nuclear Extract Kit from Active Motif (Carlsbad). Two micrograms of nuclear fractions were used to perform NFκB family (p50, p52, p65, and RelB) or NFATc1 activity assays with TransAM Transcription Factor ELISA kits (Active Motif) following the manufacturer’s protocol.

Bone Resorption Assay

Dentine slices were placed in wells of 48-well tissue culture plates. 4 x 10^4^ BMMs were seeded for each dentine slice and cells were induced to differentiate to osteoclasts. Dentine slices were washed with distilled water to remove cells, mounted on glass slides, and observed with a Zeiss LSM 5 PASCAL laser-scanning microscope (Carl Zeiss Microimaging GmbH). Resorbed area and depth were calculated by use of the Zeiss LSM Image Browser program (version 3.0 SP3).

cDNA Microarray

RNAs from MCF7 and MDA cells were prepared using the RNeasy Mini kit (Qiagen) following the manufacturer’s protocol. cDNA microarray analysis was performed by a service company (BMS, Seoul, Korea) with Human HT-12 Expression BeadChip kit (Illumina), which is designed to target more than 47,000 genes derived from the National Center for Biotechnology Information Reference Sequence. Differentially expressed gene (DEG) analysis was performed with R (version 3.4.0), using the edgeR package (version 3.20.7). After filtering genes that were expressed at low levels, the data were normalized by trimmed mean of M. DEGs were identified by ranking pairwise log2 fold change (log2FC). The heatmap was drawn by using the gplots package (version 3.0.1). Gene ontology analysis was performed by using the DAVID functional annotation suite (https://david.ncifcrf.gov), annotating sets of genes obtained from DEG analysis.

S100A4 mAb Generation

Three white leghorn chickens received biweekly injections of recombinant human S100A4 protein (Sino Biological Inc.) for 8 weeks. After the final immunization, spleen, bone marrow, and bursa of Fabricius were harvested and total RNA was isolated using TRI Reagent® (Invitrogen). First-strand cDNA synthesis was carried out using SuperScript reverse transcriptase with oligo (dT) priming (Invitrogen). Subsequently, phage display of chicken single-chain variable fragments (scFv) libraries were constructed using the pComb3XSS phagemid vector. Four rounds of bio-panning were performed. At each round, 5 x 10^6^ magnetic beads (Dynabeads M-270 epoxy, Invitrogen) coated with 1.5 μg of recombinant human S100A4 protein were used. The scFv-displaying phages were subjected to phage enzyme immunoassay. The 96-well microtiter plates (Corning) were coated overnight at 4°C with 100 ng of recombinant human S100A4 protein dissolved in coating buffer (0.1 M sodium bicarbonate, pH 8.6). The plates were blocked with 3% bovine serum albumin dissolved in PBS (w/v, PBS-B) followed by incubation with scFv-displaying phages in the culture supernatant for 2 hours at 37°C. After washing, plates were incubated with horseradish peroxidase (HRP)-conjugated mouse anti-M13 monoclonal antibody (GE Healthcare) in PBS-B for 1 hour at 37°C. Enzymatic reaction was carried out using 2,20-Azinobis [3-ethylbenzothiazoline-6-sulfonic acid]-diammonium salt (ABTS) substrate solutions (Amresco LLC) for 15 min at 37°C. At each intermittent step, plates were washed with 0.05% Tween-20 in PBS (PBST). The absorbance at 405 nm was measured using a microtiter plate reader (Labsystems AiG SL). Plasmid DNA was prepared from selected clones that have binding activity against human S100A4 recombinant protein (OD_405nm_>1.0) using a plasmid preparation kit (Qiagen). The sequence of the V_H_ and V_L_ chains of the scFv antibody was identified using the Ompseq primer (5’-AAGACAGCTATCGCGATTGCAG-3’). Sequence analysis of selected clones was performed by Macrogen (Seoul, Korea). The gene encoding 4A scFv fused to human Fc was subcloned into modified pCEP4 mammalian expression vector. Plasmid DNA was prepared using PureLink HiPure Plasmid Maxi-prep kit (Invitrogen), according to the manufacturer’s instruction. Monoclonal antibodies were produced by transient transfection of HEK293F cells (1 x 10^6^ cells/mL, Invitrogen) using polyethylenimine (Polysciences). After 5 days, supernatants from transfected cells were collected and monoclonal antibodies were purified by protein A gel chromatography (Repligen) according to the manufacturer’s instructions. Subsequently, purified antibodies were desalted against PBS using desalting column (Thermo Fisher Scientific).

**
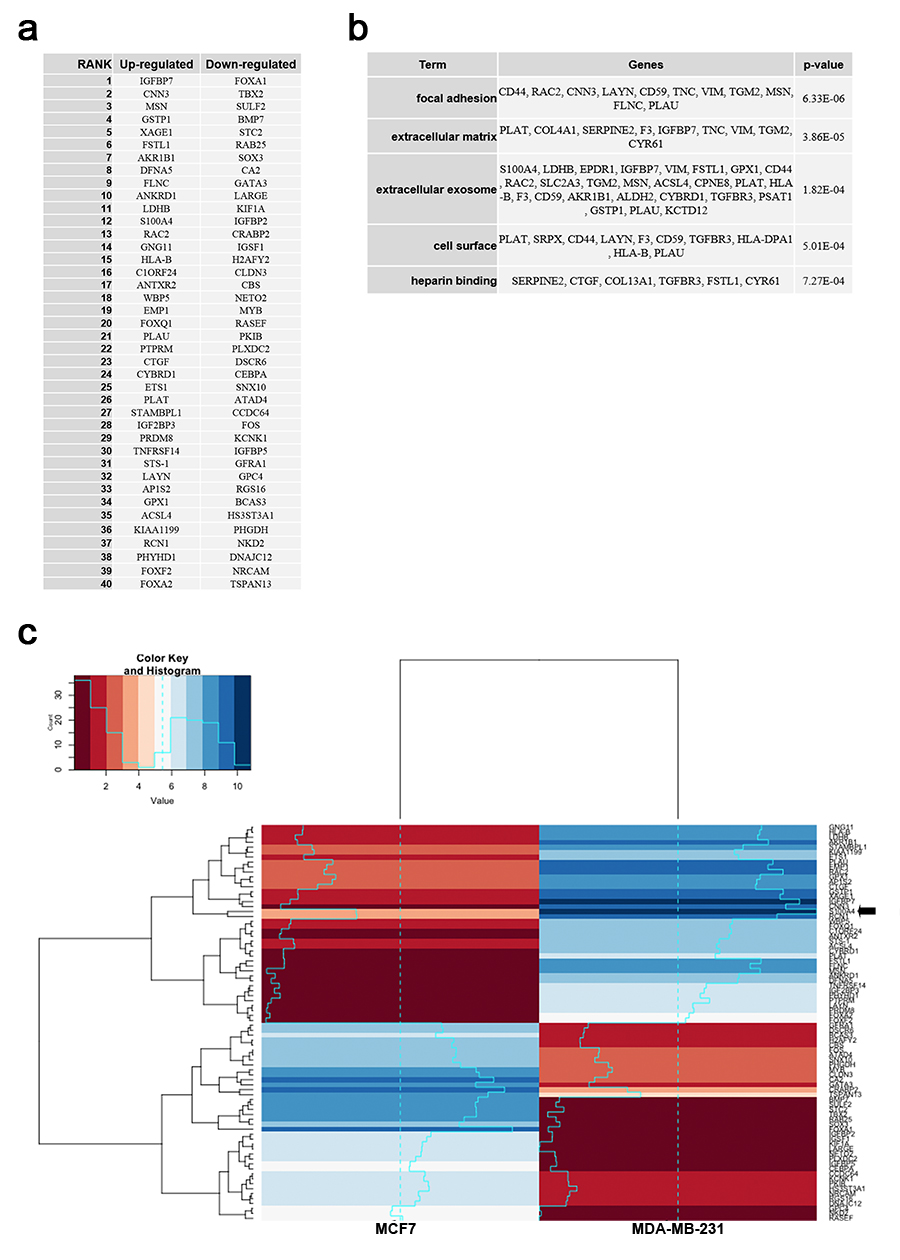
**

**Supplementary Fig. 1.** Gene expression profiling of MCF7 and MDA-MB-231 after cDNA microarray analysis. **a** Top 40 genes up-regulated or down-regulated in MDA-MB-231 in comparison with MCF7 are listed. **b** Gene ontology analysis results of top 100 genes up-regulated in MDA-MB-231 are presented with p-values. Top five categories with high significance (low p-values) are shown. **c** Hierarchical clustering of up-regulated and down-regulated top 40 genes depicted as a heat map. The arrow indicates S100A4. Blue dotted lines indicate the mean of each cell line, while blue solid lines indicate how far the gene expression deviates from the mean.


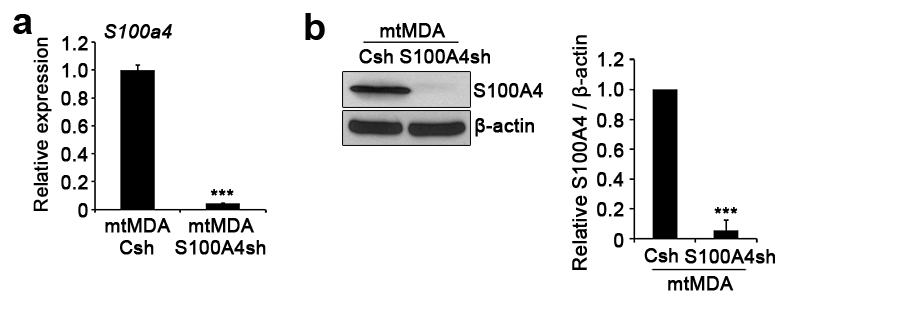


**Supplementary Fig. 2.** S100A4 knockdown in mtMDA cells. mtMDA cells were infected with lentiviruses containing control shRNA or S100A4 shRNA. The expression levels of S100A4 mRNA (**a**) and protein (**b**) were assessed by real-time PCR and Western blotting, respectively. β-Actin was included as a loading control. *n* = 3 per group. ****p* < 0.001 by unpaired two-tailed Student’s *t*-test. Data were presented as mean ± SD.


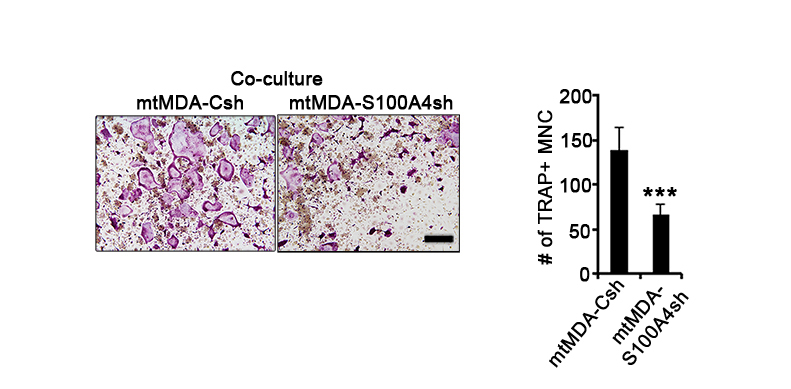


**Supplementary Fig. 3.** S100A4 knockdown in mtMDA reduces osteoclastogenesis-stimulating activity. Pre-OCs were co-cultured with mtMDA-Csh or mtMDA-S100A4sh cells before TRAP-staining (left). TRAP^+^ MNC were quantified (right). *n* = 5 per group. ****p* < 0.001 by unpaired two-tailed Student’s *t*-test. Data were presented as mean ± SD. Scale bars, 200 μm.


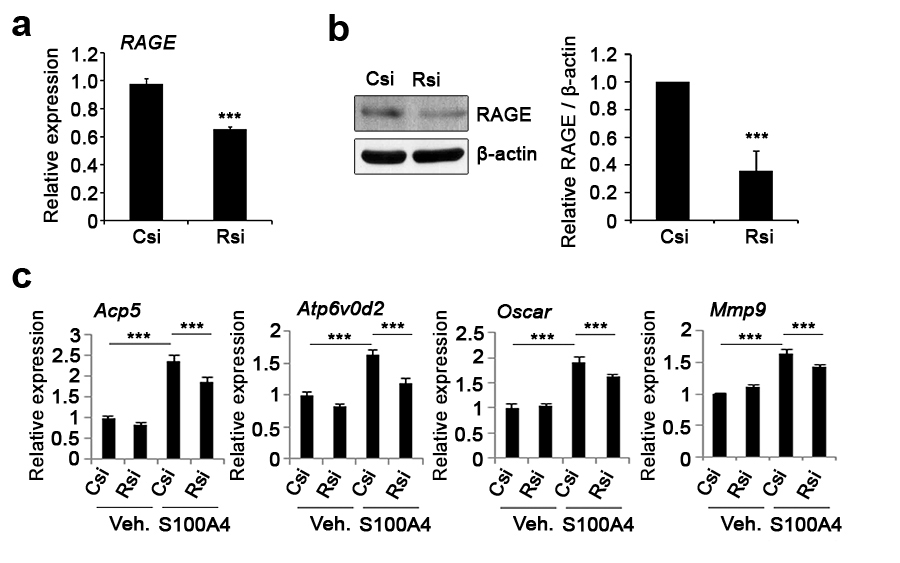


**Supplementary Fig. 4.** Knockdown of RAGE abrogates S100A4-induced expression of osteoclast marker genes. **a and b** Pre-OCs were transfected with control (Csi) or RAGE (Rsi) siRNA. The mRNA and protein levels of RAGE were assessed by real-time PCR (**a**) and Western blotting (**b**). *n* = 3 per group. ****p* < 0.001 by unpaired two-tailed Student’s *t*-test. **c** The knockdown cells were treated with vehicle (Veh.) or rS100A4 (1 μg/mL) for 1 day. Real-time PCR analyses to assess expression levels of *Acp5* (TRAP), *Atp6v0d2*, *Oscar*, and *Mmp9* were performed. *n* = 3 per group. ****p* < 0.001 by two-way ANOVA with post hoc Tukey’s test. All data were presented as mean ± SD.

**
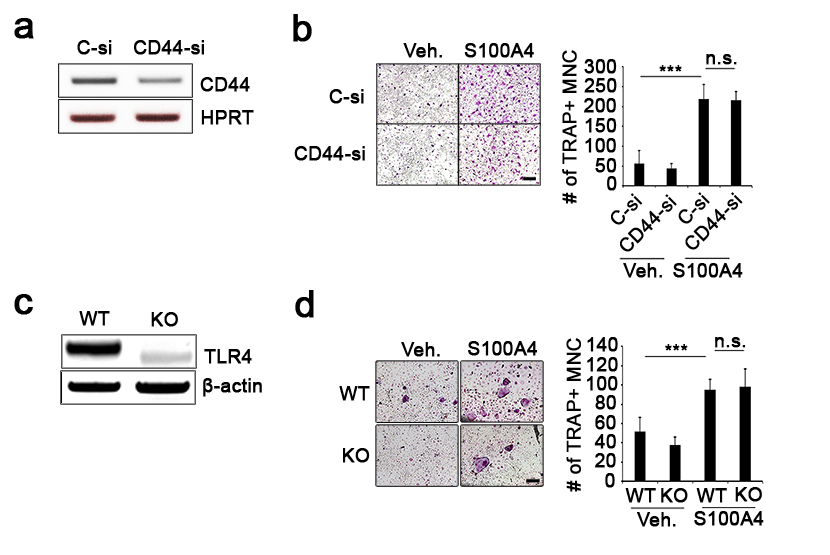
**

**Supplementary Fig. 5.** S100A4 enhancement of osteoclastogenesis is not mediated by CD44 and TLR4. **a and b** Pre-OCs with control (C-si) or CD44 (CD44-si) knockdown were treated with vehicle (Veh.) or rS100A4 (1 μg/mL) for 2 days. The efficiency of knockdown was determined by RT-PCR (**a**). Cells were stained for TRAP (**b**, left) and TRAP^+^ MNC were counted (**b**, right). *n* = 5 per group. *** *p* < 0.001 for Veh/C-si vs. S100A4/C-si and *p* = 0.875 for S100A4/C-si vs. S100A4/CD44-si by two-way ANOVA with post hoc Tukey’s test. **c and d** Pre-OCs generated from wild-type (WT) and TLR4 knockout (KO) BMMs were treated with vehicle (Veh.) or rS100A4 (1 μg/mL) for 2 days. The expression of TLR4 was determined by RT-PCR (**c**). Cells were stained for TRAP (**d**, left) and TRAP^+^ MNC were counted (**d**, right). *n* = 5 per group. *** *p* < 0.001 for Veh/WT vs. S100A4/WT and *p* = 0.747 for S100A4/WT vs. S100A4/KO by two-way ANOVA with post hoc Tukey’s test. All data were presented as mean ± SD. n.s., not significant. Scale bars, 100 μm.


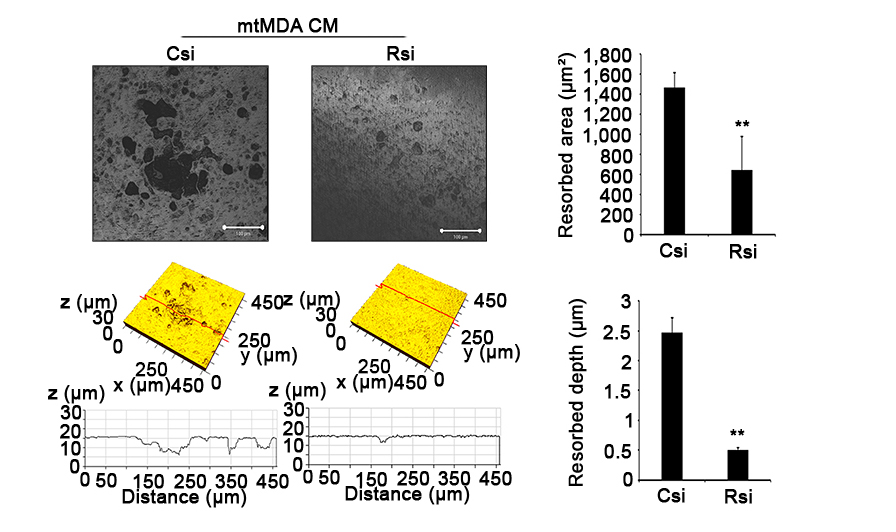


**Supplementary Fig. 6.** RAGE knockdown inhibits mtMDA-stimulation of bone resorption. Pre-OCs with control (Csi) or RAGE (Rsi) knockdown cultured on dentine slices were treated with CM from mtMDA. Dentine slices were analyzed by confocal microscopy. Representative images of dentine surfaces (left) and values of resorbed area and depth of pits are presented (right). *n* = 8 per group. ** *p* < 0.01 by unpaired two-tailed Student’s *t*-test. All data were presented as mean ± SD. Scale bars, 100 μm.


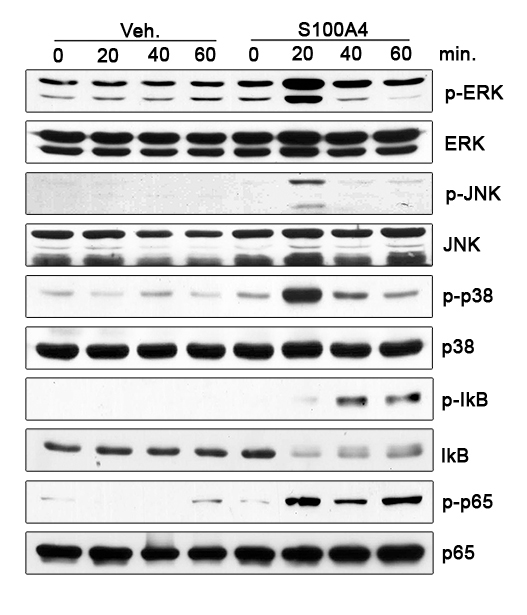


**Supplementary Fig. 7.** MAPKs and NFκB are activated by S100A4. Pre-OCs were stimulated with rS100A4 (1 μg/mL) for the indicated times. Western blotting with whole cell lysates with indicated antibodies are shown.


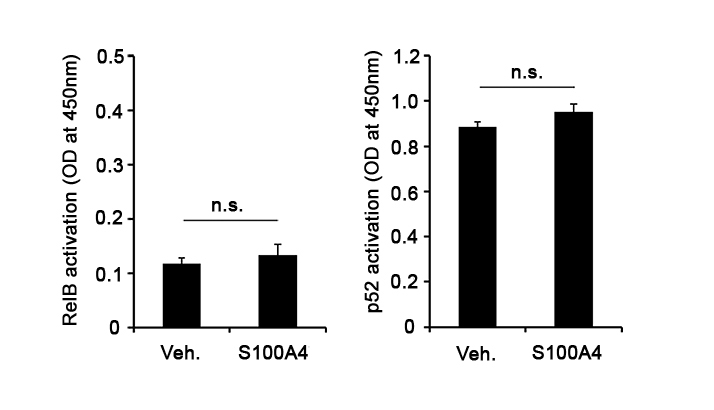


**Supplementary Fig. 8.** The noncanonical NFκB pathway is not activated by S100A4. Pre-OCs were stimulated with vehicle (Veh.) or mouse rS100A4 (2 μg/mL) for 1 hour. Results of transcription factor assays for RelB and p52 are shown. *n* = 3 per group. *p* = 0.258 for RelB and 0.052 for p52 by unpaired two-tailed Student’s *t*-test. n.s., not significant. All data were presented as mean ± SD.


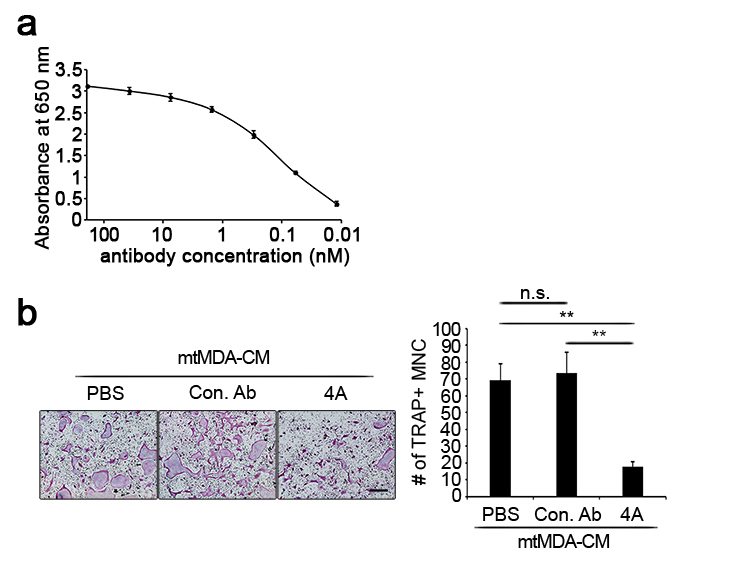


**Supplementary Fig. 9.** Anti-S100A4 mAb 4A decreases in vitro osteoclastogenesis. **a** Binding activity of S100A4 mAb clone 4A for human rS100A4 was measured with various concentrations of the purified Ab. **b** Pre-OCs were cultured with mtMDA CM (30%) together with a control antibody (Con. Ab, 30 μg/mL), anti-S100A4 (4A, 30 μg/mL), or equal volume of PBS for 2 days. TRAP-stained images (left) and quantification of TRAP^+^ MNC (right) are shown. *n* = 3 per group. *p* = 1.000 for PBS vs. Con. Ab, and ***p* < 0.01 for both PBS vs. 4A Ab and Con. Ab vs. 4A Ab by one-way ANOVA with post hoc Tukey’s test. n.s., not significant. All data were presented as mean ± SD. Scale bars, 200 μm.
